# Self-Adherent Biodegradable Gelatin-Based Hydrogel Electrodes for Electrocardiography Monitoring

**DOI:** 10.3390/s20205737

**Published:** 2020-10-09

**Authors:** Yechan Lee, Sang-Gu Yim, Gyeong Won Lee, Sodam Kim, Hong Sung Kim, Dae Youn Hwang, Beum-Soo An, Jae Ho Lee, Sungbaek Seo, Seung Yun Yang

**Affiliations:** Department of Biomaterials Science (BK21 Four Program), College of Natural Resources & Life Science, Pusan National University, Miryang 50463, Korea; yclee071@gmail.com (Y.L.); sg.yim0425@gmail.com (S.-G.Y.); 22jungbi@gmail.com (G.W.L.); damsoho@gmail.com (S.K.); khs@pusan.ac.kr (H.S.K.); dyhwang@pusan.ac.kr (D.Y.H.); anbs@pusan.ac.kr (B.-S.A.); james007din@pusan.ac.kr (J.H.L.); sbseo81@pusan.ac.kr (S.S.)

**Keywords:** hydrogel electrode, electrocardiography (ECG), gelatin, PEDOT:PSS, bioelectronic device

## Abstract

Patch-type hydrogel electrodes have received increasing attention in biomedical applications due to their high biocompatibility and conformal adherence. However, their poor mechanical properties and non-uniform electrical performance in a large area of the hydrogel electrode should be improved for use in wearable devices for biosignal monitoring. Here, we developed self-adherent, biocompatible hydrogel electrodes composed of biodegradable gelatin and conductive polymers for electrocardiography (ECG) measurement. After incorporating conductive poly(3,4-ethylenedioxythiophene):poly(4-styrenesulfonate) (PEDOT:PSS) into gelatin hydrogels crosslinked by natural crosslinkers (genipin), the mechanical properties and electrical conductivity of the hydrogel electrodes were improved and additionally optimized by adjusting the amounts of crosslinker and PEDOT:PSS, respectively. Furthermore, the effect of dimethyl sulfoxide, as a dopant, on the conductivity of hydrogels was investigated. The gelatin-based, conductive hydrogel patch displayed self-adherence to human skin with an adhesive strength of 0.85 N and achieved conformal contact with less skin irritation compared to conventional electrodes with a chemical adhesive layer. Eyelet-type hydrogel electrodes, which were compatible with conventional ECG measurement instruments, exhibited a comparable performance in 12-lead human ECG measurement with commercial ECG clinical electrodes (3M Red Dot). These self-adherent, biocompatible, gelatin-based hydrogel electrodes could be used for monitoring various biosignals, such as in electromyography and electroencephalography.

## 1. Introduction

Since cardiovascular diseases have been recognized as the leading cause of patient death globally, medical electrodes enabling the measurement of biological signals, such as in electrocardiography (ECG), have been extensively researched [1,2]. Integrating medical electrodes into wearable devices in particular makes it possible to remotely monitor patients’ health, thereby providing essential information as a means to diagnose diseases in their early stages [3,4].

A medical electrode device or patch generally consists of an electrode, a cable connector, and an adhesive part for effectively measuring a biosignal [5]. Electrodes are conventionally prepared by using polarizable metals such as platinum and gold or non-polarizable electrodes such as Ag/AgCl. The polarized electrode displaces current through the loading and unloading of the electrode interface, while current flows in the non-polarizable electrode pass directly through an oxidation-reduction reaction at the electrode–electrolyte interface [6]. A metal electrode is suitable for neural recoding, requiring sharp tissue penetrating ability [7], but it might not be effective in surface ECG applications due to the applicant’s possible allergic response to metals and poor contact causing motion artifacts [8,9]. The Ag/AgCl-gel electrode is widely used for cardiac monitoring in clinical settings. However, the chemical adhesive layer in conventional electrodes could potentially cause skin irritation following long-term use [10].

Recently, flexible and conductive hydrogel electrodes have been extensively studied due to their high biocompatibility and improved electrode performance in biological environments compared to conventional metal electrodes [11,12,13,14,15]. In order to improve the mechanical and electrical properties of hydrogel electrodes, a method of blending the hydrogel with conducting polymers has been used [16]. Conducting polymers, such as polypyrrole (PPy), polyaniline (PANI), and poly (3,4-ethylenedioxythiophene) (PEDOT), carbon nanotubes (CNTs) and graphene have been incorporated into a hydrogel network by various fabrication routes [11,17,18,19,20]. A common method for the fabrication of a hybrid conducing polymer/hydrogel electrode is the polymerization of conducting polymers in preformed hydrogel matrices (or solutions) following the introduction (or mixing) of monomers of the conducting polymers. These two steps are needed because the conducting polymers have poor solubility in an aqueous hydrogel-forming solution. Poly(3,4-ethylenedioxythiophene) polystyrene sulfonate (PEDOT:PSS), which is a water-dispersible form of PEDOT doped with the water-soluble PSS, has been used to fabricate a conductive hydrogel [21,22,23]. The conductive hydrogel film containing PEDOT:PSS showed good electrochemical properties with long-term stability while the hydrogel required an additional adhesive layer for tissue adhesion and was prepared by synthetic polymers with low biocompatibility [21,24,25].

Recently, there have been several studies to increase the biocompatibility of hydrogels by using biopolymers such as polysaccharides or proteins [26,27]. Gelatin is denatured and hydrolyzed collagen is found in most connective tissues such as skin, tendon, and bone. It is widely used in pharmaceutical and biomedical applications as hard and soft capsule material and as a tissue engineering scaffold due to its biocompatibility, biodegradability, and cost-effectiveness. As gelatin has a typical sol-gel transition temperature of 30 °C, an aqueous gelatin solution prepared above 60 °C can be converted to a solid hydrogel form by cooling the solution to room temperature [28,29]. The weak mechanical properties and the low thermal stability of gelatin hydrogels limit their practical use; however, it has been demonstrated that the mechanical and thermal properties of gelatin can be improved through chemical or physical crosslinking methods [30,31].

In this study, we developed biocompatible, self-adhesive hydrogel electrodes for ECG measurements prepared from gelatin crosslinked with genipin, as a natural water-soluble crosslinking agent with less toxicity. The mechanical and electrical properties of the gelatin-based hydrogel electrode were improved by incorporating a water-dispersible, conducting polymer (PEDOT:PSS). In addition, we investigated the doping effect of dimethyl sulfoxide (DMSO) on optimizing the performance of gelatin-based hydrogel electrodes. The adhesive properties and biocompatibility of the PEDOT:PSS/gelatin hydrogel were investigated using experimental animals. ECG monitoring (12-lead) was performed using both the gelatin-based hydrogel electrodes developed in this study and commercial electrodes (Red Dot 2237, 3M).

## 2. Materials and Methods

### 2.1. Materials

Porcine skin gelatin was purchased from Sigma-Aldrich (St. Louis, MO, USA). Genipin was purchased from Wako chemical (Osaka, Japan). PEDOT:PSS aqueous solution with a solid content of 1.37 wt% was purchased from Sooyang chemtec (Yesan, Korea). Dimethyl sulfoxide (DMSO) was purchased from Mallinckrodt Baker (Phillipsburg, NJ, USA). Red Dot™ foam monitoring electrode 2237 was purchased from 3M (Maplewood, MN, USA).

### 2.2. Fabrication of PEDOT:PSS/Gelatin Hydrogel Electrodes

**Figure 1:**
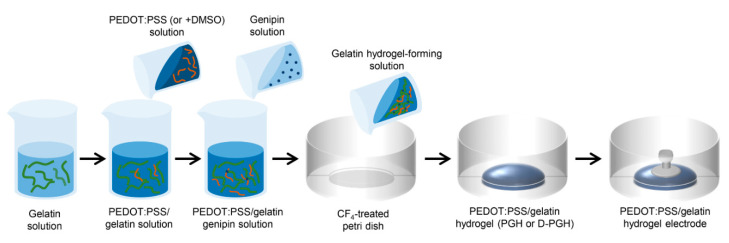
Schematic presentation of the preparation procedure for gelatin-based hydrogel electrodes.

The fabrication procedure of gelatin-based hydrogel electrodes is schematically shown in Figure 1. An aqueous solution of porcine skin gelatin (Sigma-Aldrich) was prepared in a 37 °C water bath by stirring for about 1 h. Then, a PEDOT:PSS aqueous solution was mixed with the gelatin solution to achieve a total solid content of 10% (*w*/*v*). The PEDOT:PSS solution at pH 7 was used prior to mixing with gelatin in order to prevent the rapid gelation of gelatin in acidic conditions. Subsequently, PEDOT:PSS was added to the gelatin solution at weight ratios of 0:10, 0.1:10, 0.5:10, 1:10, and 2:10 [13]. After forming a homogeneous mixture of PEDOT:PSS/gelatin, genipin, used as a crosslinker of gelatin, was added to the PEDOT:PSS/gelatin solution in a 1:100 genipin-to-gelatin weight ratio. One milliliter of hydrogel-forming solution was poured onto 6 cm petri dishes and crosslinked at 25 °C for 24 h. DMSO, used as a dopant of PEDOT:PSS, was added and mixed in the PEDOT:PSS/gelatin solution prior to the addition of the genipin solution. The DMSO concentrations were 1, 3, 5, and 10 wt% in hydrogel-forming solutions. The samples used in this study are shown in Table 1. For ECG measurements with a commercially available instrument, eyelet-type PEDOT:PSS-incorporated gelatin hydrogel (PGH) electrodes were fabricated by inserting an aluminum button into partially crosslinked PGH films for 10 min. The button was fixed after complete crosslinking for 24 h.

### 2.3. Mechanical Tests

To confirm the effects of different mixing ratios of PEDOT:PSS/gelatin on mechanical properties, hydrogel mixtures for each ratio were prepared. Mechanical strength was measured using a mechanical tester (5000 H, AND) in the form of a dog bone (ASTM D1708) at 25 °C [32]. The dog bone-shaped specimens were prepared by die-cutting a gelatin hydrogel film with a thickness of 1 mm. The hydrogel was then stretched by a 5 kg load cell until it was broken at a constant rate of 10 mm/min. An average value of seven samples was taken. The mechanical properties of the hydrogel such as tensile strength, Young’s modulus, toughness, and elongation were calculated from stress–strain curves.

### 2.4. Measurement of Electrical Properties

Changes in electrical properties were confirmed by the content of PEDOT:PSS. Gelatin hydrogel film resistance was measured using a surface resistance meter with 2 probes (SRT 557, DESCO). In order to confirm the uniformity of electrical resistance in the sample, five points in the sample were measured, and the uniformity of electrical resistance between samples was also confirmed. The resistance at the center of the sample and four positions 1 cm away from the center of the sample was measured.

### 2.5. Adhesion Tests 

To measure the adhesion of the hydrogel, a hydrogel was attached to a human arm. D-PGH films were fabricated as a circular hydrogel with a diameter of 22 mm. The adhesive forces were measured with a digital force measurement gauge (SLD 5FGN, SPC Technology) on the skin surface. After the hydrogel was fixed on a push–pull tensioner, the hydrogel was preloaded for 10 s and then slowly removed from the skin. All participants gave their informed consent before they participated in the study.

### 2.6. Biocompatibility Tests 

To confirm the biocompatibility of skin electrodes used in this study, skin irritation was assessed after attaching it to the skin of the experimental animals for 24 h. The animal experiment protocol used for this study was approved by the Institutional Animal Care and Use Committee (IACUC; approval number PNU-2015-0950) of Pusan National University. Eight-week-old male Sprague–Dawley (SD) rats (200–300 g) were purchased from Samtako Bio Korea Co. (Osan, Korea), Ltd. and fed a standard irradiated chow diet (Samtako Bio Korea Co.) and water throughout the experimental process. During the experiment, all rats were grown in a specific pathogen-free state under a strict light cycle (lights on at 08:00 h and off at 20:00 h) with a constant temperature of 23 ± 2 °C and 50 ± 10% relative humidity. The rats were housed at the Pusan National University-Laboratory Animal Resources Center accredited by the Korea Ministry of Food and Drug Safety in accordance with the Laboratory Animal Act (Accredited Unit Number: 000231) and AAALAC International according to the National Institutes of Health guidelines (Accredited Unit Number: 001525).

### 2.7. ECG Measurements 

To evaluate biosignal sensing performance, 12-lead ECG measurements were conducted using gelatin (D_0.01_-PGH_0.1_) hydrogel electrodes; commercial electrodes (Red Dot 2237, 3M) were used as control. The electrodes were attached to the participant’s body at the standard 10 positions and then connected to a commercial ECG machine (AT-101, Schiller) to record surface potentials associated with the electrical activity of the heart. The filtered and amplified analog ECG waveforms were converted to digital signals following transmission to a personal computer. All participants gave their informed consent before they participated in the study.

### 2.8. Statistical Analysis

Data were analyzed using Student’s *t*-test with Excel software (Microsoft Corp., Redmond, WA, USA) and expressed as the mean ± standard deviation (SD) of independent experiments. A *p* < 0.05 indicates statistical significance.

## 3. Results and Discussion

### 3.1. Fabrication of Conductive Gelatin-Based Hydrogels

**Figure 2:**
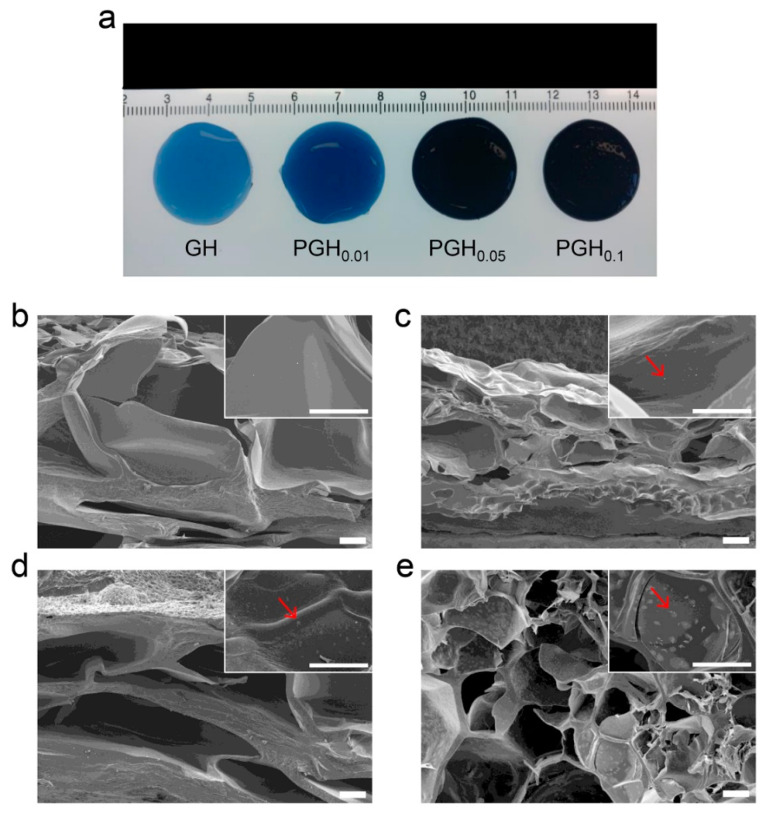
(**a**) Photograph showing the genipin-crosslinked hydrogel (GH) and poly(3,4-ethylenedioxythiophene):poly(4-styrenesulfonate) (PEDOT:PSS)-containing gelatin hydrogels (PGHs) with different mixing ratios of gelatin and PEDOT:PSS. (**b**–**e**) Cross-sectional SEM images of freeze-dried hydrogels: (**b**) GH, (**c**) PEDOT:PSS-incorporated gelatin hydrogel (PGH)_0.01_, (**d**) PGH_0.05_, and (**e**) PGH_0.1_. Scale bars = 100 μm.

As shown in the fabrication procedure of PGHs (Figure 1), gelatins and PEDOT:PSSs were mixed in the aqueous solution and then genipin was added to prepare hydrogel-forming solutions. The gelation proceeded by a two-step chemical reaction between the primary amines of gelatin and genipin for 24 h at 25 °C [30]. This procedure allows a uniform dispersion of conductive PEDOT:PSS polymers in the crosslinked gelatin network. When the mixing ratio of PEDOT:PSS and gelatin exceeded 2:10, the solutions became too viscous, possibly due to molecular interactions between PEDOT:PSS and gelatin. For better processability, we fixed the concentration of crosslinkers and controlled the amount of PEDOT:PSS in the hydrogel-forming solutions to optimize the material properties. While the genipin-crosslinked gelatin hydrogel (GH) was blue, PEDOT:PSS-containing gelatin hydrogels (PGHs) gradually turned to darker shades of blue as the amount of PEDOT:PSS increased (Figure 2a). Since the PGH electrode can be prepared by one-step molding process, the gelatin-based formulation provides a simple and cost-effective fabrication method of electroconductive hydrogels for bioelectronic applications. To investigate the internal structure of PGHs, scanning electron microscope (SEM) measurements were conducted with cross-sectioned freeze-dried hydrogels. While the pore size of hydrogel did not change with the addition of PEDOT:PSS, the PGHs showed the presence of distributed particles on the hydrogel pore walls compared to the GHs without PEDOT:PSS (Figure 2b–e). These dispersed PEDOT:PSS particles offer a pathway for charge conduction within the hydrogel network [33,34]. 

### 3.2. Mechanical Properties of Gelatin-Based Hydrogel Electrodes

**Figure 3:**
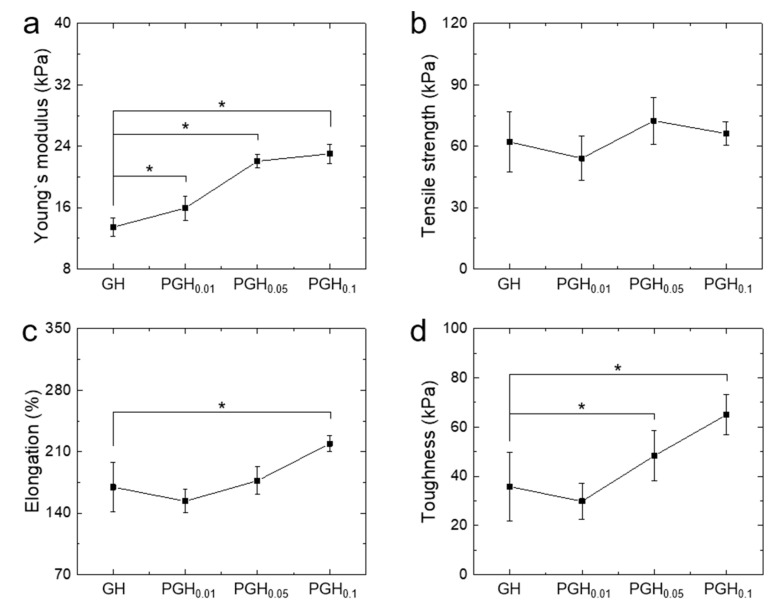
Mechanical properties of gelatin-based hydrogel electrodes obtained in tensile tests. (**a**) Young’s modulus, (**b**) tensile strength, (**c**) elongation, and (**d**) toughness (n = 7). * *p* < 0.05.

The mechanical properties of PGH electrodes were investigated. Although the addition of PEDOT did not affect the tensile strength of the hydrogels, their stiffness, elongation, and toughness were improved with increasing PEDOT:PSS amounts (Figure 3). Interestingly, PGH_0.1_ showed a 30% higher elongation and twice the toughness compared with GH (Figure 3d). This was possibly due to multiple networks produced by inter-crosslinking of gelatins and the entanglement between gelatin and PEDOT:PSS chains [35]. 

Since hydrogel-based skin electrodes require proper strength and flexibility to achieve conformal contact in dynamic stretching/contraction conditions, PGH_0.1_ would be a great candidate for use as electrode material. 

### 3.3. Electrical Conductivity of Hydrogel Electrodes

**Figure 4:**
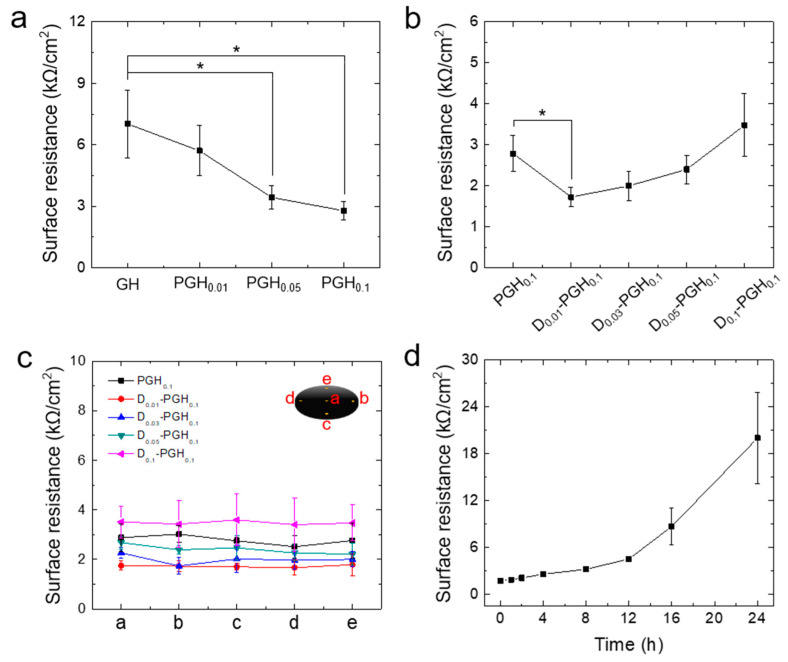
(**a**) Surface resistance of gelatin-based hydrogel electrodes according to mixing ratio of PEDOT:PSS and gelatin. (**b**) Surface resistance of dimethyl sulfoxide (DMSO)-doped PGH_0.1_ with different amounts of DMSO. (**c**) Surface resistance of DMSO-doped PGH_0.1_ measured at different 5 positions. (**d**) Surface resistance change of D_0.01_-PGH_0.1_ depending on drying time at ambient conditions (25 °C and 35% humidity) (n = 7). * *p* < 0.05.

ECG electrodes analyze the electrical signal by measuring on the surface of the body currents generated in the sinoatrial node of the heart. For ECG measurement, hydrogel electrodes must be sufficiently electro-conductive to transmit biosignals. The electrical conductivity of PGH electrodes was measured using a surface resistance meter. The surface resistance of hydrogel electrodes decreased as the composition of PEDOT:PSS increased. Notably, the standard deviation of surface resistance in obtained samples was also reduced, indicating an improved sensing reliability (Figure 4a). Compared to GH and PGH_0.1_ surface resistance was lowered from 7.5 to 2.5 kΩ because as the amount of PEDOT:PSS increased, the number of polymers capable of transmitting electrical signals also increased, and it was confirmed that PEDOT:PSS was uniformly dispersed in the hydrogel (Figure 2e). To further improve the electrical conductivity of gelatin-based hydrogel electrodes, we added DMSO, which has been known to enhance the performance of PEDOT:PSS, in order to lower the surface resistance of PGH electrodes [36]. The addition of DMSO into the PEDOT:PSS solutions could rearrange the conductive PEDOT polymer chains, resulting in an increased conductivity due to more chain interactions [37]. In addition, DMSO is advantageous for the preparation of hydrogel electrodes because of its high mixability and low evaporation rate in PEDOT:PSS formulations. To investigate the effect of DMSO on electrical conductivity, DMSO was added at 1, 3, 5, and 10 wt% of PGH_0.1_-forming solutions, which have the lowest surface resistance. For skin application, low concentrations (<10 wt%) of DMSO are known to be safe to use [38]. The SEM images showed uniformly distributed PEDOT:PSS in the hydrogel which DMSO was added (Figure S1).

The 1 wt% DMSO-doped PGH electrodes (D_0.01_-PGH_0.1_) showed a 50% lower surface resistance compared to PGH electrodes without DMSO (PGH_0.1_) but a further addition of DMSO resulted in reduced conductivity (Figure 4b). Interestingly, DMSO addition improved the reliability (less standard deviation) and uniformity of electrical conductivity in the entire area of the D_0.01_-PGH_0.1_ (Figure 4c). This improved electrical conductivity in hydrogels could be a result of the increased phase separation of PEDOT:PSS and the expansion of PEDOT chains due to DMSO doping, thereby facilitating electron transfer between PEDOT:PSS chains [39]. However, the DMSO doping effect was not significant at high DMSO concentrations (>3 wt%), possibly due to the limited molecular transformation of PEDOT chains [40,41]. The mechanical properties of the hydrogel electrodes were not significantly affected by the addition of DMSO (Figure S2). As the electrical performance of hydrogel-based electrodes can be affected by surface dehydration, we investigated surface resistance changes in the hydrogel electrodes with time at ambient conditions (25 °C and 35% humidity) to evaluate the possibility of long-term use. As water evaporated from the hydrogels (D_0.01_-PGH_0.1_) over time, the weight of the hydrogels gradually decreased and was not significantly changed after 24 h (Figure S3). The surface resistance slowly increased with a rate of 0.23 kΩ/h at early time points (up to 12 h) but sharply increased after 12 h. Since typical ECG measurements are completed within 1 h, the hydrogel electrodes used in this study would be reliable and effective for ECG measurement. For long-term biosignal monitoring, we will apply an air-impermeable cap on top of the gel to minimize water evaporation in future work.

### 3.4. Skin Adhesion Tests Using Self-Adherent Hydrogel Electrodes

**Figure 5:**
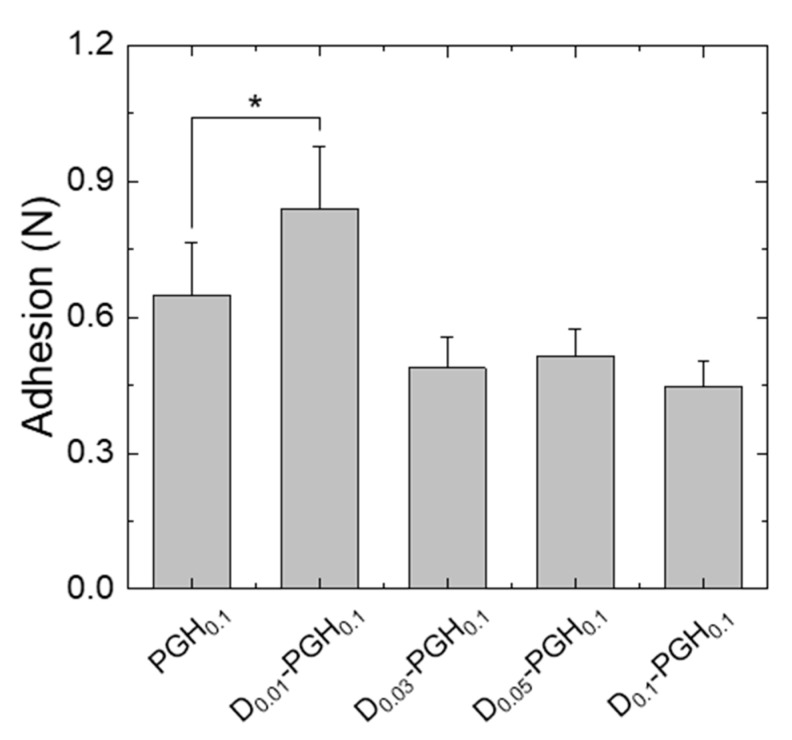
Skin adhesion of PGHs with different compositions (n = 5). * *p* < 0.05.

To achieve a stable and long-term monitoring of biosignals, proper adhesion of the electrodes to the target tissue is essential. We tested the adhesion strength of conductive gelatin-based hydrogel (PGH) electrodes after applying them to the participant’s forearm. The PGHs (22-mm disc) showed seamless contact and stable adhesion to the skin without the need for an additional adhesive layer. The small addition of DMSO to the PGH improved skin adhesion (0.22 ± 0.03 N/cm^2^ for D_0.01_-PGH_0.1_) while the adhesion of PGHs decreased after the addition of DMSO above a certain level (Figure 5). Considering the overall performance (mechanical properties, electrical conductivity, and skin adhesion) of hydrogels electrodes, we selected the D_0.01_-PGH_0.1_ formulation to prepare ECG electrodes.

### 3.5. ECG Measurement Using Biocompatible Self-Adherent Hydrogel Electrodes 

Prior to ECG measurement, animal experiments were carried out to confirm the biocompatibility of hydrogel electrodes. Gelatin-based hydrogel electrodes (D_0.01_-PGH_0.1_) and commercial electrodes (3M Red Dot) were attached to the shaved dorsal skin of rats through the application of a stretchable adhesive (Coban, 3M) for 24 h (Figure S4). Compared with commercial electrodes, D_0.01_-PGH_0.1_ electrodes closely adhered to the curved surface of the skin (Figure S4a,c). After 24 h, the hydrogel and the commercial electrodes were removed from the skin of the rats, and the skin tissues of the rats were examined. No skin irritation or rash was observed on the skin tissues of rats with D_0.01_-PGH_0.1_ application (Figure S4b). However, swelling occurred on the skin of the rat with the commercial electrode application (Figure S4d).

**Figure 6:**
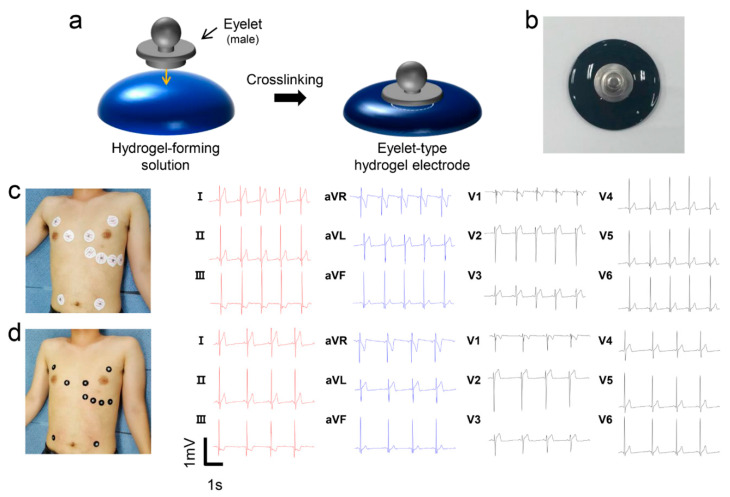
(**a**) Schematic illustration showing the fabrication of eyelet-type surface electrodes using conductive hydrogel-forming solutions. (**b**) Photograph of the D_0.01_-PGH_0.1_ hydrogel electrode used for electrocardiography (ECG) measurement. (**c**,**d**) Waveforms obtained from 12-lead ECG measurement using (**c**) commercial electrodes (Red Dot 2237, 3M) and (**d**) D_0.01_-PGH_0.1_ hydrogel electrodes.

To evaluate the performance of conductive gelatin hydrogels in ECG measurement, eyelet-type surface electrodes were prepared for compatibility with a conventional ECG measurement instrument. Eyelet-type hydrogel electrodes (22-mm disc) were fabricated by placing eyelets into partially crosslinked hydrogel-forming solutions (Figure 6a). As previously demonstrated, optimal formulations (D_0.01_-PGH_0.1_ solutions) were used to prepare the hydrogel electrodes for good electric conductivity and skin adhesion. The D_0.01_-PGH_0.1_ electrode exhibited firm adhesion between the eyelet and the hydrogel (Figure 6b). ECG measurements were performed using hydrogel electrodes following conventional 12-lead ECG operational instructions after placing 10 electrodes on the surface of the participant’s chest [42]. Commercial ECG electrodes with foam tape and sticky gel were used for reference measurement. After applying the electrodes to the 12-lead ECG measurement positions, ECG tracing was recorded for 5 min following 5 min of relaxation. The D_0.01_-PGH_0.1_ surface electrode displayed the capacity to replicate standard 12-lead ECG waveforms obtained from reference electrodes (Figure 6c,d). Interestingly, the R-wave amplitude in II, III, and aVF, which could be used in the diagnosis of heart diseases, was similarly observed without noise when compared to the reference electrode [43]. In addition, the D_0.01_-PGH_0.1_ electrode showed a better signal to noise ratio than the GH electrodes without conducting polymers (Figure S5). A visual inspection after ECG measurement revealed slight skin irritation and flushing in the area where the form adhesive peeled off. However, no skin damage was observed after the use of the hydrogel electrodes. Compared to commercial electrodes (Red Dot 2237, 3M), the gelatin hydrogel electrodes (D_0.01_-PGH_0.1_) exhibited a comparative ECG measurement performance, offering sufficient skin adhesion without additional adhesive and minimal skin damage. Based on this result, the hydrogel electrode could be used for arrhythmias since it provides a uniform measurement signal without causing inflammation even after attachment for a long time [44]. 

## 4. Conclusions

In this study, we developed self-adherent, biocompatible gelatin-based hydrogel electrodes incorporating conductive PEDOT:PSS, providing conformal skin adhesion and uniform electrical conductivity. By controlling the amount of PEDOT:PSS polymers, the mechanical properties of the conductive gelatin hydrogel were optimized. Moreover, the electrical performance (resistance variation and surface resistance of hydrogel electrodes) was improved by adding DMSO that acts as a dopant. The conductive hydrogels exhibited a stable electrical performance during dehydration at ambient conditions without a significant decrease in electrical conductivity. Importantly, the gelatin hydrogel showed self-adherence to skin tissue with conformal contact and minimal tissue damage. Its biocompatibility was confirmed through animal experiments, and the hydrogel electrodes using biopolymers were found to have no skin irritation and swelling when applied to animal skin compared to the commercial electrodes. In addition, flushing was not felt in the electrodes using hydrogel with more than 90% water. During 12-lead ECG measurement with eyelet-type hydrogel electrodes, the detection performance of ECG signals was similar to that of the reference electrode (Red Dot 2237, 3M). Considering the ease of the molding process with conductive hydrogel-forming solutions, large-scale, cost-effective fabrication is possible. Moreover, by introducing nano- or micro-structures onto the surface of hydrogel electrodes, the adhesive performance of the electrodes to target tissues can be improved [45,46]. Self-adherent hydrogel electrodes with good biocompatibility and flexibility could be integrated into motion detection sensors and into wearable sensors for medical monitoring.

## Figures and Tables

**Table 1:** Gelatin hydrogels used in the experiments.

Hydrogel	Composition
GH	Gelatin/Genipin (1:0.01)
PGH_0.01_	PEDOT:PSS/Gelatin/Genipin (0.01:1:0.01)
PGH_0.05_	PEDOT:PSS/Gelatin/Genipin (0.05:1:0.01)
PGH_0.1_	PEDOT:PSS/Gelatin/Genipin (0.1:1:0.01)
D_0.01_-PGH_0.1_	DMSO(0.01)–PEDOT:PSS/Gelatin/Genipin (0.1:1:0.01)
D_0.03_-PGH_0.1_	DMSO(0.03)–PEDOT:PSS/Gelatin/Genipin (0.1:1:0.01)
D_0.05_-PGH_0.1_	DMSO(0.05)–PEDOT:PSS/Gelatin/Genipin (0.1:1:0.01)
D_0.1_-PGH_0.1_	DMSO(0.1)–PEDOT:PSS/Gelatin/Genipin (0.1:1:0.01)

## Supplementary Materials

The following are available online at https://www.mdpi.com/1424-8220/20/20/5737/s1, Figure S1: Cross-sectional SEM images of freeze-dried DMSO-doped hydrogel electrodes. (a) D_0.01_-PGH_0.1_, (b) D_0.03_-PGH_0.1_, (c) D_0.05_-PGH_0.1_, and (d) D_0.1_-PGH_0.1_. Scale bars = 100 μm; Figure S2: Mechanical properties of DMSO-doped PGHs. (a) Young’s modulus, (b) tensile strength, (c) elongation, and (d) toughness. n = 7; Figure S3: Change in the weight of hydrogel electrodes with time; Figure S4: Biocompatibility test of D_0.01_-PGH_0.1_ using SD rats. Adhesive image of the (a) hydrogel and (c) 3M Red dot electrode at shaved dorsal skin of the rats. (b) Dorsal skin of the rats after removal of the hydrogel and (d) 3M Red dot electrode after 24 h application; Figure S5. Overlay graphs of waveforms obtained from 12-lead ECG measurement using commercial electrodes (black line) and D_0.01_-PGH_0.1_ hydrogel electrodes (red line); Figure S6: Waveforms obtained from 12-lead ECG measurement using GH hydrogel electrodes. The arrow indicates noise in measurement.
