# Supplementary material for: Self-Adherent Biodegradable Gelatin-Based Hydrogel Electrodes for Electrocardiography Monitoring
Source: Sensors (Basel). 2020 Oct 9;20(20):5737. doi: 10.3390/s20205737 (PMC7601183; doi:10.3390/s20205737)
Supplement: Supplementary file 1 [file sensors-20-05737-s001.pdf]

Supplementary materials

Self-adherent biodegradable gelatin-based hydrogel electrodes for electrocardiography monitoring

Yechan Lee†, Sang-Gu Yim†, Gyeong Won Lee, Sodam Kim, Hong Sung Kim, Dae Youn Hwang, Beum-Soo An, Jae Ho Lee, Sungbaek Seo and Seung Yun Yang*

† Equal contribution

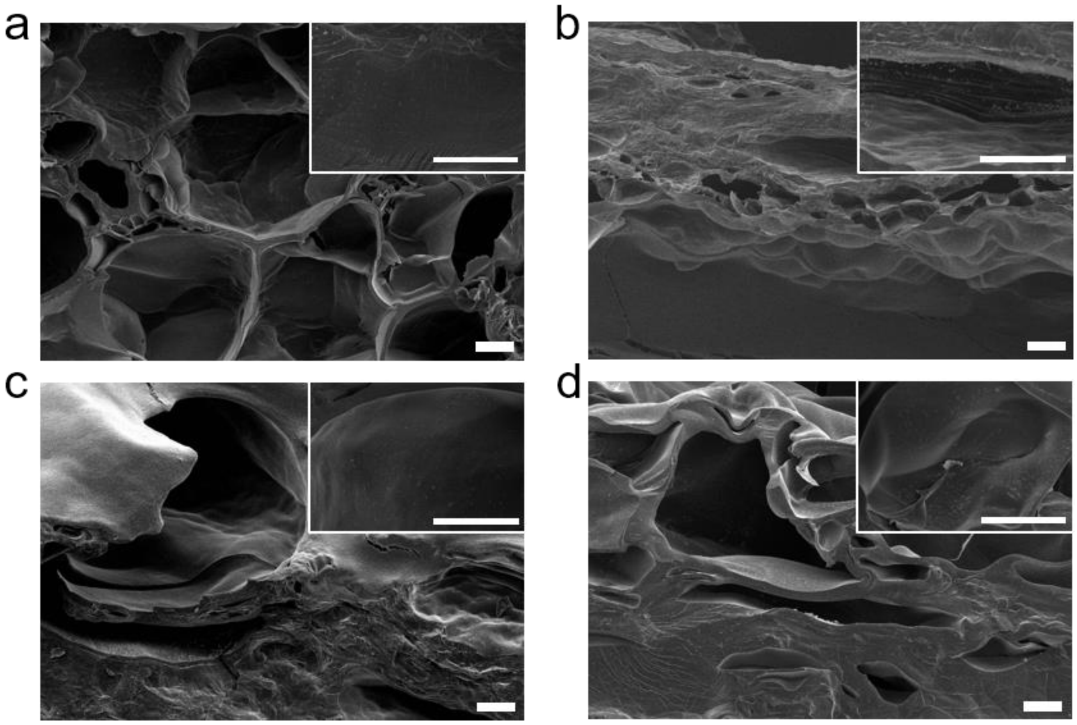

Figure S1. Cross-sectional SEM images of freeze-dried DMSO-doped hydrogel electrodes. (a) $D_{0.01}$-$PGH_{0.1}$, (b) $D_{0.03}$-$PGH_{0.1}$, (c) $D_{0.05}$-$PGH_{0.1}$, and (d) $D_{0.1}$-$PGH_{0.1}$. (scale bars = 100 μm)

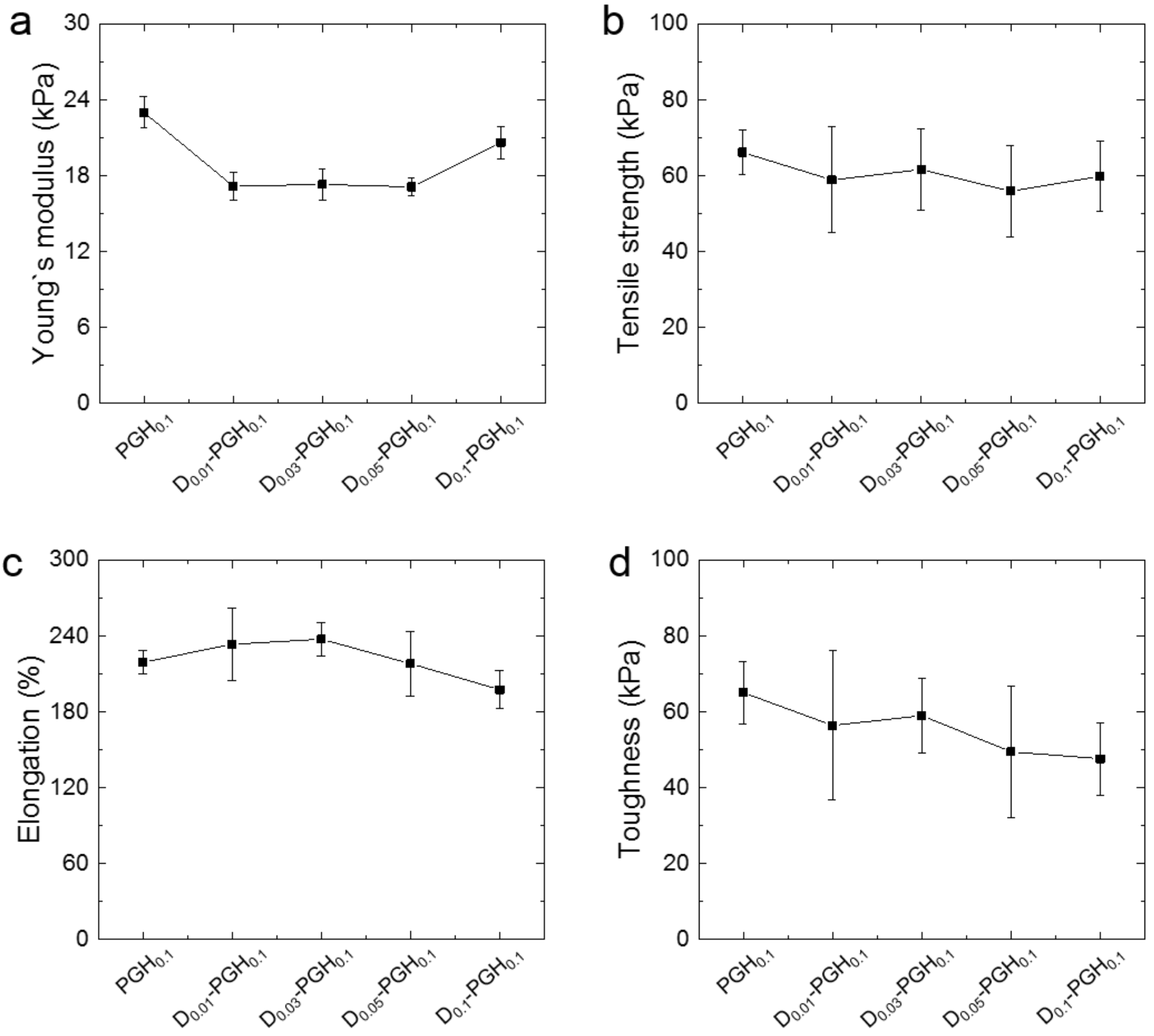

Figure S2. Mechanical properties of DMSO-doped PGHs. (a) Young's modulus, (b) tensile strength, (c) elongation, and (d) toughness. (n = 7)

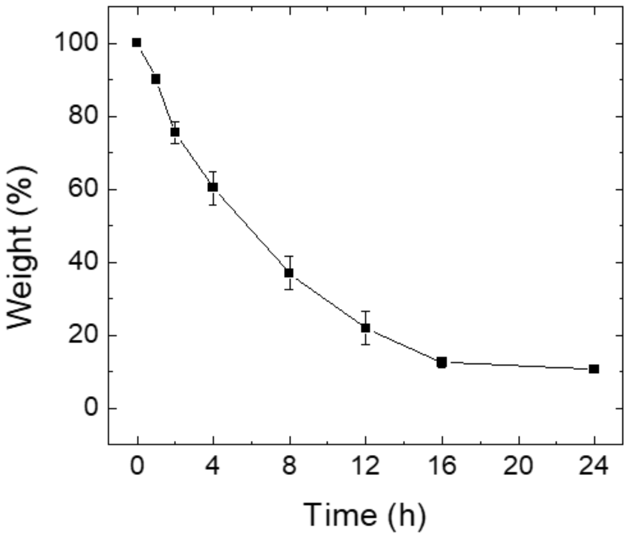

Figure S3. Change in the weight of hydrogel electrodes with time.

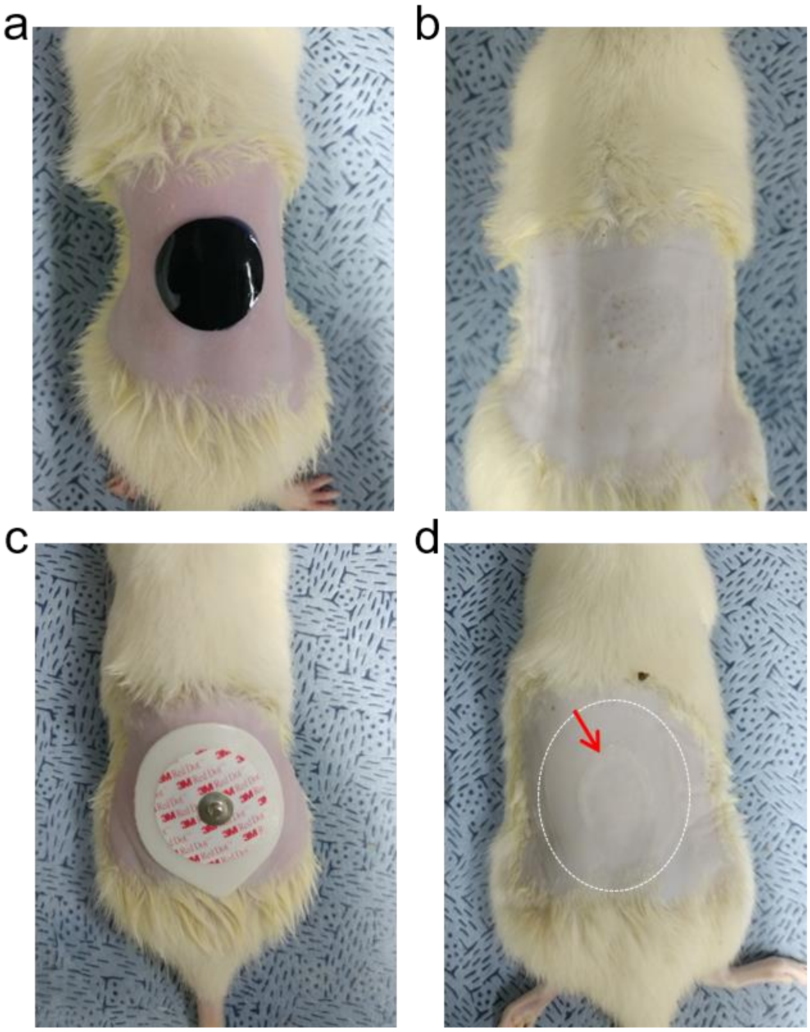

Figure S4. Biocompatibility test of $D_{0.01}$-$PGH_{0.1}$ using SD rats. Adhesive image of the (a) hydrogel and (c) 3M Red dot electrode at shaved dorsal skin of the rats. (b) Dorsal skin of the rats after removal of the hydrogel and (d) 3M Red dot electrode after 24 h application.

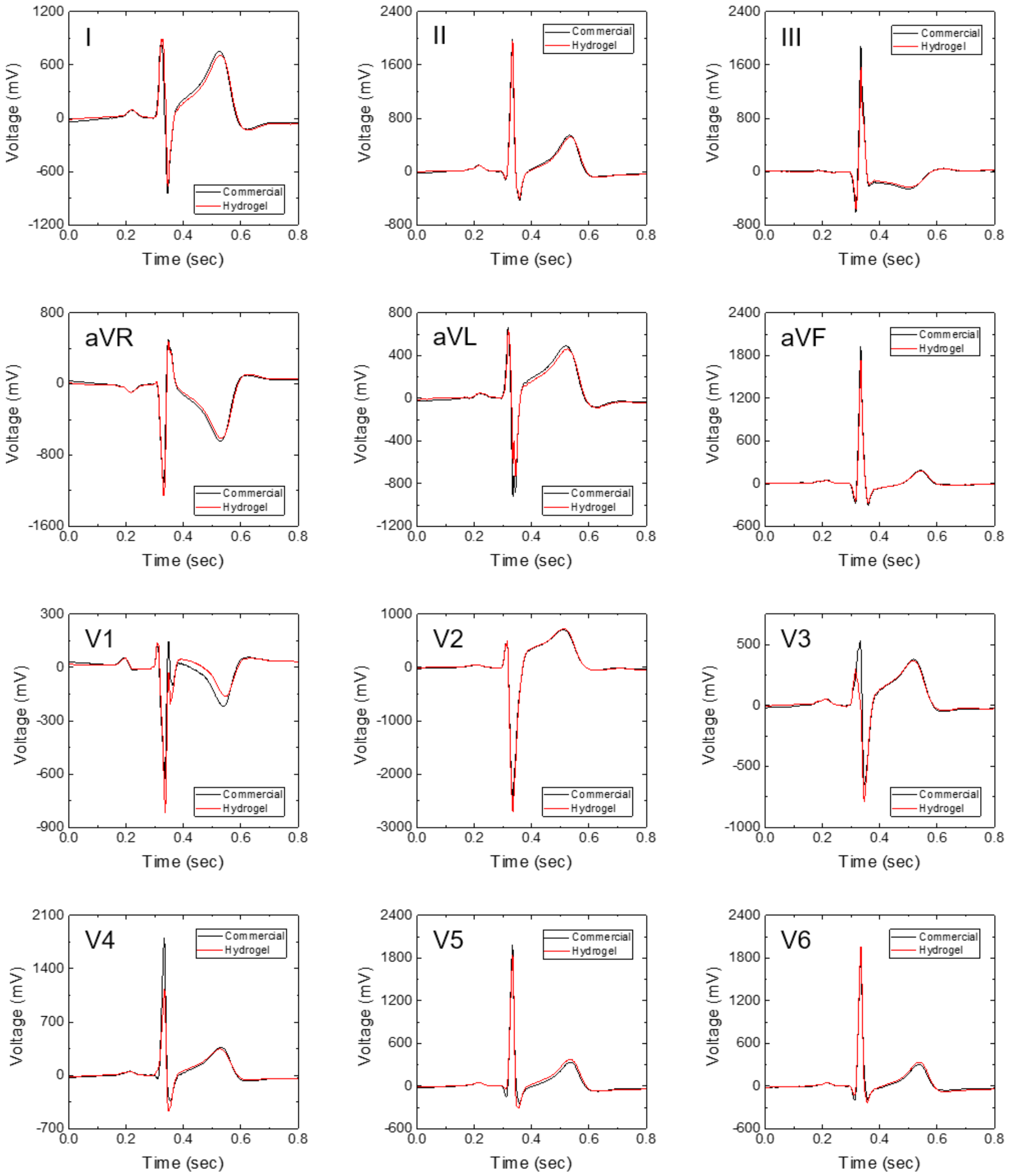

Figure S5. Overlay graphs of waveforms obtained from 12-lead ECG measurement using commercial electrodes (black line) and D0.01-PGH0.1 hydrogel electrodes (red line).

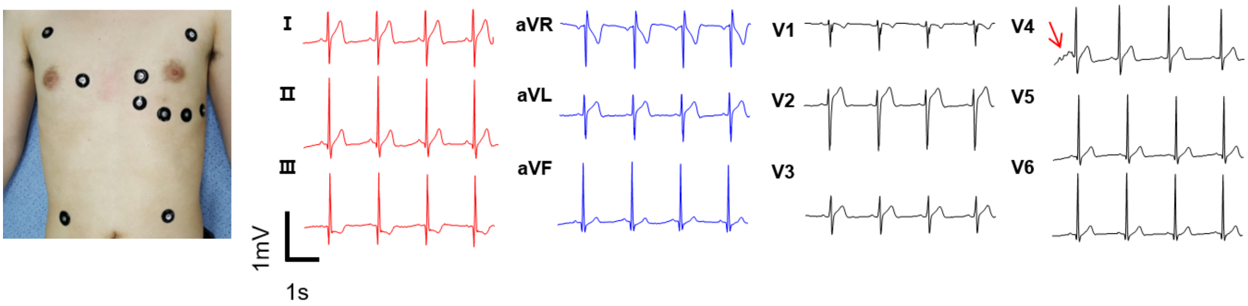

Figure S6. Waveforms obtained from 12-lead ECG measurement using GH hydrogel electrodes. The arrow indicates noise in measurement.
